# The EANM guideline on radioiodine therapy of benign thyroid disease

**DOI:** 10.1007/s00259-023-06274-5

**Published:** 2023-07-03

**Authors:** Alfredo Campennì, Anca M. Avram, Frederik A. Verburg, Ioannis Iakovou, Heribert Hänscheid, Bart de Keizer, Petra Petranović Ovčariček, Luca Giovanella

**Affiliations:** 1 Department of Biomedical and Dental Sciences and Morpho-Functional Imaging, Unit of Nuclear Medicine, University of Messina, Messina, Italy; 2 Departments of Radiology and Medicine, MetroHealth Hospital, Case Western Reserve University, Cleveland, OH USA; 3 Department of Radiology & Nuclear Medicine, Erasmus MC, Rotterdam, The Netherlands; 4 Academic Department of Nuclear Medicine, University Hospital AHEPA, School of Medicine, Aristotle University of Thessaloniki, Thessaloniki, Greece; 5 Academic Department of Nuclear Medicine, General Hospital Papageorgiou, Aristotle University of Thessaloniki, Thessaloniki, Greece; 6 Department of Nuclear Medicine, University Hospital Würzburg, Würzburg, Germany; 7 Department of Radiology and Nuclear Medicine, University Medical Centre Utrecht, Utrecht, The Netherlands; 8 Department of Oncology and Nuclear Medicine, University Hospital Center Sestre Milosrdnice, Zagreb, Croatia; 9 School of Medicine, University of Zagreb, Zagreb, Croatia; 10 Clinic for Nuclear Medicine, Ente Ospedaliero Cantonale, Imaging Institute of Southern Switzerland, Bellinzona, Switzerland; 11 Clinic for Nuclear Medicine, University Hospital and University of Zurich, Zurich, Switzerland

**Keywords:** Graves’ disease, Toxic nodular goiter, Non-toxic goiter, RAI therapy, Fixed activity, Dosimetry

## Abstract

This document provides the new EANM guideline on radioiodine therapy of benign thyroid disease. Its aim is to guide nuclear medicine physicians, endocrinologists, and practitioners in the selection of patients for radioiodine therapy. Its recommendations on patients’ preparation, empiric and dosimetric therapeutic approaches, applied radioiodine activity, radiation protection requirements, and patients follow-up after administration of radioiodine therapy are extensively discussed.

## Preamble

The European Association of Nuclear Medicine (EANM) is a professional non-profit medical association that facilitates communication worldwide among individuals pursuing clinical and research excellence in nuclear medicine. The EANM was founded in 1985.

These guidelines are intended to assist practitioners in providing appropriate nuclear medicine care for patients. They are not inflexible rules or requirements of practice and are not intended, nor should they be used, to establish a legal standard of care.

The ultimate judgment regarding the propriety of any specific procedure or course of action must be made by medical professionals taking into account the unique circumstances of each case. Thus, there is no implication that an approach differing from the guidelines, standing alone, is below the standard of care. To the contrary, a conscientious practitioner may responsibly adopt a course of action different from that set out in the guidelines when, in the reasonable judgment of the practitioner, such course of action is indicated by the condition of the patient, limitations of available resources, or advances in knowledge or technology subsequent to publication of the guidelines.

The practice of medicine involves not only the science but also the art of dealing with the prevention, diagnosis, alleviation, and treatment of disease. The variety and complexity of human conditions make it impossible to always reach the most appropriate diagnosis or to predict with certainty a particular response to treatment. Therefore, it should be recognized that adherence to these guidelines will not ensure an accurate diagnosis or a successful outcome.

All that should be expected is that the practitioner will follow a reasonable course of action based on current knowledge, available resources, and the needs of the patient to deliver effective and safe medical care. The sole purpose of these guidelines is to assist practitioners in achieving this objective.

## Autoimmune hyperthyroidism (Graves’ disease) and non-autoimmune toxic nodular goiter/toxic multinodular goiter

### Introduction

Radioactive iodine-131 (RAI) therapy has been used for the treatment of hyperthyroidism for more than 80 years since Doctor Saul Hertz successfully administered the first therapeutic activity of radioiodine-131 (I-131) to a patient with Graves’ disease (GD) in early 1941.

Since then, millions of patients worldwide have received RAI therapy for definitive treatment of hyperthyroidism due to either autoimmune (i.e., GD) or non-autoimmune toxic (multi)-nodular goiter (i.e., TMNG/TNG, respectively) [1]. Graves’ disease (GD) [Morbus Basedow in German-speaking countries] and autonomously functioning thyroid nodules (i.e., toxic nodular goiter, TNG; toxic multinodular goiter, TMNG) are the most common causes of hyperthyroidism (i.e., GD 70–80% of cases, TNG/TMNG 20–30% of cases).

RAI therapy has also been employed as an alternative to surgery for the treatment of patients affected by large non-toxic nodular goiter (NTG) to achieve a significant reduction of gland volume [2–5].

The use of RAI therapy has drastically diminished the number of patients requiring surgery for definitive treatment of hyperthyroidism, thus reducing both the risk of surgical complications (e.g., recurrent and superior laryngeal nerve palsy, hypoparathyroidism, hemorrhage) and healthcare costs [1]. RAI is the classic agent used for the diagnosis and treatment of benign thyroid disease based on sodium-iodine symporter (NIS) expression in normal thyroid tissue and its widespread medical use represents the first application of the theragnostic concept in medicine, remaining to this day the cornerstone of radionuclide therapy.

The accumulated clinical experience over the past eight decades has proven that RAI therapy is safe and highly effective, with current literature offering several schools of thought and differing insights on how to approach this treatment. Since the publication of the prior edition of this guideline [6], these insights have been significantly enriched. For instance, a recent meta-analysis of various dosimetry data from studies on RAI therapy for GD showed a dose–response relationship between the delivered radiation absorbed dose to the thyroid and the therapeutic outcome (i.e. rates of hyper-, eu-, and hypothyroidism after RAI treatment) [7]. Therefore, the current document presents an updated guideline for the use of RAI in the treatment of benign thyroid disease with the aim to guide/support nuclear medicine physicians, endocrinologists, and practitioners in selecting patients for RAI therapy. In addition, recommendations on patients’ preparation, strategy approaches (i.e., empiric vs. dosimetric therapy), radioiodine activity regimes, radiation protection requirements, and follow-up management are duly discussed.

### Radionuclides of interest for thyroid imaging and therapy

Functional diagnostic imaging of patients with benign thyroid disease involves the use of ^99m^Tc-pertechnetate (Na[^99m^Tc]Tc0_4_) and radioiodine-123 (Na[^123^I]I) due to their specificity for the sodium iodide symporter (NIS). Details on uptake mechanisms, pharmacokinetics, biodistribution, clinical indications of different tracers, and imaging procedures are out of the scope of the present guideline; readers are referred to the EANM practice guideline/SNMMI procedure standard for RAIU and thyroid scintigraphy [8].

The therapeutic radioisotope Na[^131^I]I has a half-life of 8.02 days and decays by β-electrons emission with a maximum energy of the main transition of 0.61 MeV, and an average energy release per decay of 0.192 MeV. The average range in the tissue of the β-particle is 0.4 mm. In addition, Na[^131^I]I emits an electromagnetic photon with a principal gamma-ray of 364 keV. Based on these physical characteristics, Na[^131^I]I was the first theragnostic radiopharmaceutical: its gamma-photon emission was used to observe and quantify iodine distribution and kinetics within the thyroid gland, while its β-particle emission (β-radiation) was used to obtain the therapeutic effect.

### General considerations for RAI therapy

The rationale underlying RAI therapy is based on the ability of follicular thyroid cells to take up Na[^131^I]I, in the same fashion as stable iodine (^127^I) absorbed from dietary sources.

The therapeutic use of Na[^131^I]I has two main applications:

(i) To treat hyperthyroidism in patients with GD or TNG/TMNG;
(ii) To reduce the volume of the whole gland (e.g., GD or non-toxic goiter (NTG)) or hyperfunctioning nodule(s).

Patients affected by the above conditions referred for RAI therapy should be evaluated and eventually treated with I-131 according to international guidelines [6, 9] and local medical standards. Patients with absolute contraindications to RAI therapy must be promptly identified and excluded from this therapeutic procedure. The patients who qualify for RAI therapy require consultation with a nuclear medicine specialist to discuss specific information and preparation for this procedure based on the patient’s specific diagnosis, age, and comorbidities. Informed consent should be obtained before RAI therapy administration following national procedural recommendations. Detailed written instructions to reduce radiation exposure and the risk of contamination of family members and the general public must be given to patients before release after therapy.

#### RAI therapy for benign thyroid disease methods: patient preparation, dosimetry, and radiation protection requirements

Patient candidates for RAI therapy must be evaluated by a nuclear medicine specialist for discussing the indications, logistics, and expectations of RAI treatment. This consultation will also include a review of current medications and specific dietary preparation instructions in anticipation for RAI administration, as well as checking for current and/or prior use of iodine-containing products (Table 1] with resultant decreased radioiodine uptake (RAIU) in the thyroid gland which can reduce RAI therapy efficacy [1, 6, 10–12]. Notably, RAIU testing and RAI therapy should be performed under the same condition (i.e., preparation protocol, medications).

**Table 1:** Thyroid drugs and iodide-containing substances that can reduce radioiodine thyroid uptake

Type of medication	Recommended time of withdrawal
Water-soluble intravenous radiographic contrast agents	6–8 wk*, assuming normal renal function
Lipophilic intravenous radiographic contrast agents	3–6 mo^#^
Thyroxine	3–4 wk*
Triiodothyronine	10–14 d^§^
Antithyroid drugs:	
Methimazole Propylthiouracil	2–5 d^§^ before RAI therapy 2–8 wk* if RAI therapy is performed by fixed-activity method (4–7 d^§^ if RAI therapy is performed after personalized dosimetric approach)
Nutrition supplements containing iodide	7–10 d^§^
Kelp, agar, carrageenan, Lugol solution	2–3 wk*, depending on iodide content
Saturated solution of potassium iodide	2–3 wk*
Topical iodine (e.g., surgical skin preparation)	2–3 wk*
Amiodarone	3–6 mo^#^ or longer

Adapted from SNMMI Procedure Standard 2012

^*§*^*d* days; *wk weeks; ^*#*^*mo* months

Pregnancy is an absolute contraindication for RAI treatment. In all fertile females, it can be excluded by a careful history collection and demonstrated by obtaining hCG serum testing just before RAI administration (if not feasible within 72 h maximum). If there is a suspicion of early pregnancy (not detectable by beta-HCG) according to anamnestic data, RAI therapy should be postponed. Patients’ preparation protocols may differ depending on different thyroid diseases and treatment goals. Details are reported in the specific sections.

#### Radiation protection requirements

Any medical examination and treatment involving radiation exposure to patients, treating medical personnel, or members of the public should be carried out in accordance with the safety standards on radiation protection laid down in Chapter VII of the Council Directive 2013/59/Euratom [13].

With regard to the protection of patients treated with radiation, Article 56 of the directive is relevant: “For all medical exposure of patients for radiotherapeutic purposes, exposures of target volumes shall be individually planned and their delivery appropriately verified taking into account that doses to non-target volumes and tissues shall be as low as reasonably achievable and consistent with the intended radiotherapeutic purpose of the exposure.” [13].

Although the member states of the European Union were obliged to transpose the directive into national law, the requirement for individually planned treatments has not generally been adopted for nuclear medicine therapies. Irrespective of the present recommendations for action, medical practitioners must comply with the national formulations of the applicable laws, particularly when measurements of patient-specific biokinetics and retention time functions and verification of the administered radiation absorbed doses can be performed.

Depending on national regulations for treatments with unsealed radioactive agents, it may be necessary to have the treatment performed by specially trained personnel in a dedicated therapy ward with appropriately shielded rooms and suitable radiation protection equipment. Usually, such a ward has forced ventilation with adequate air exchange and is connected to a sewage system for the collection of contaminated wastewater. Treatment should be provided by a team of physicians, nurses, technologists, and medical physics experts with a high level of competence and a clear definition of tasks and responsibilities, considering legal requirements. Written instructions should be available for the handling of radioactive materials, waste, and treated patients. Adequate monitoring of personnel for exposure to direct radiation and/or contamination is required in line with national and regulatory requirements.

#### Dosimetry for therapy planning and dose verification

Dosimetry in conjunction with RAI therapy for benign thyroid disease can be performed either pre-therapeutically for determining the necessary Na[^131^I]I administered activity which will deliver the radiation absorbed dose to the target tissue that is expected to be effective in the particular type of disease, or post-therapeutically for evaluating the energy dose absorbed in tissue. The energy dose D from self-irradiation of a radioiodine accumulating target mass M, which can be a substructure of the thyroid gland such as a hyper-functioning adenoma or the whole thyroid gland such as in Graves’ disease or goiter, is given by the product of the mean energy Ē deposited in M per decay of ^131^I and the number of decays represented by the time integral of the activity A(t) in M. A(t) is often written as product Aa· RIU(t), where Aa is the administered activity and RIU(t) the radioiodine uptake, i.e., the fraction of the administered activity in M at a given time t after the administration. The activity necessary to achieve a specified radiation absorbed dose D in the target mass M is [14]:

1 $${A}_{a}\left[MBq\right]=\frac{1}{\overline{E} }\cdot \frac{M\left[g\right]\cdot D\left[Gy\right]}{\underset{0}{\overset{\infty }{\int }}RIU\left(t\right)dt\left[d\right]}$$

The constant value recommended in [14] for the mean energy deposited in the target tissue per decay of ^131^I,

2 $$\overline{E }=2.808\frac{Gy\cdot g}{MBq\cdot d}$$

which was calculated for thyroid with *M* = 20 g, will produce results with adequate accuracy for most of the patients. Mean energy deposition is about 5% higher due to increased photon absorption in goiters with *M* = 90 g.

Complete dosimetry requires measurements of the mass of the target tissue to be treated and the time function RIU(t) of iodine uptake in this mass. The activity required to achieve a specified absorbed dose is linearly dependent on the target mass. Any error in the mass estimate induces a correspondent error in the activity or absorbed dose calculations. For reliable dosimetry, morphological imaging should be used to measure the target mass. Two-dimensional ultrasound is inferior to three-dimensional ultrasound [15–17]; however, it is easy to perform, cost-effective, and reliable in most patients [17–20], and it is recommended in routine diagnostics. Computed tomography (CT) [21], magnetic resonance imaging (MRI) [20], and positron emission tomography (PET) [22] have been described as suitable, but as they are overly expensive and demanding, might however be considered in rare cases of large goiters with substernal extension. Planar and tomographic scintigraphies have limited spatial resolution and are sufficiently accurate only for target masses greater than approximately 20 g [20].

In particular, planar scintigraphy for mass estimation should be reserved for patients in whom the target tissue cannot be delineated or completely visualized in ultrasound, which is considerably more accurate for volume measurement. The measurement must then be performed after administration of Na[^99m^Tc]TcO_4_ using a camera system with high spatial resolution and, after suitable contour finding, the volume must be estimated from the projection area of the accumulating gland [20]. Before planar scintigraphy is applied for volume determination, phantom studies and comparisons on patients with both scintigram and ultrasound measurement must be performed to verify that the contour finding and the equation for calculating volume from area provide sufficiently correct results for the gamma camera and acquisition parameters actually used.

From planar images with high spatial resolution, usually obtained by ultrasound, the mass M of a substructure such as a thyroid lobe or a nodule can be estimated from the lengths of the longest diameter A and 2 orthogonal axes B and C in the transverse section with the largest area as *M* = A,·B,·C/2, with A, B, and C, in centimeters and M in grams [14]. For large structures approximately shaped like an ellipsoid, the error of the mass estimate is typically less than 20%. Higher errors are possible for very small nodules, if the target mass is not clearly circumscribed in imaging, in patients with toxic goiters with several nodules or disseminated disease, or if the morphological imaging does not match the scintigraphically documented activity distribution. Morphological images used to deduce a mass estimate should always be compared to a Na[^99m^Tc]TcO_4_ or Na[^123^I]I scintigram in order to verify that the measured volume corresponds to the accumulating target tissue.

The time integral of the uptake function determines the number of decays and thus the absorbed dose in the target mass per unit of activity administered. At least 3 uptake measurements are required to calculate the uptake function. Recommended time points for measurements are 4–6 h after the administration during the initial increase of uptake, after 1–2 days shortly after the time of maximum uptake, and after 5–8 days when the decrease of uptake allows for a reliable estimate of the effective half-life of the activity in the target tissue [14]. Making use of theoretical compartment model considerations [23, 24], the number of uptake assessments can be reduced to two measurements obtained after 1–2 days and after 5–8 days, or even to only one measurement obtained after 5–8 days with minor loss of accuracy [14, 23–26]. To ensure reliable uptake measurements and consistent dose calculations, it is recommended to follow the standard operational procedures on pre-therapeutic dosimetry provided in [14].

#### Simplifying activity regimes

If instead of a late measurement of RIU(t), only the maximum uptake is estimated from an early assessment after time *te*, the measured value is not representative of the time integral in the denominator of Eq. (1]. The activity to be administered in such an approach should be calculated by [14]:

3 $${A}_{a}\left[MBq\right]=\frac{0.714}{\overline{E} }\cdot \frac{M\left[g\right]\cdot D\left[Gy\right]}{RIU\left({t}_{e}\right)\cdot {2}^{{t}_{e}/{T}_{est}}\cdot {T}_{est}\left[d\right]}$$

with a reasonable estimate *T*_est_ of the effective half-life of the activity in the gland. As recommended in [14], a fixed value *T*_est_ = 5.5 days might be used for all patients or disease-specific half-lives if they are known to be representative of the respective patient population. With *T*_est_ = 5.5 days, the absorbed dose is a factor of 1.24 higher than expected if the actual effective half-life is 7 days and factors of 1.31 and 1.65 lower for 4 and 3 days actual effective half-life, respectively. Accuracy increases with the increasing time of the measurement after the administration of Na[^131^I]I. The potential for error is lower with a measurement after 2 days than with a measurement after one day or earlier.

Activity dosage linear to the mass M (g) without any pretherapeutic assessment of iodine kinetics in the target tissue assumes that the time integral over RIU(t) matches a predefined value. Possible overdosing is limited by the theoretically possible maximum value of the integral, which results from almost complete retention in M and decay with the physical half-life of ^131^I. Significant underdosing and thus ineffective treatment is possible in the case of unexpectedly low uptake and/or half-life.

The highest deviations from an adequate target dose D are possible when fixed activities are administered without considering any influence of thyroid tissue mass, radiation absorbed dose, and iodine kinetics on treatment outcome. Increased rates of residual or recurrent disease are expected with large masses and rapid iodine kinetics. Substantial overdosage is possible with small masses, e.g., a fixed activity of 555 MBq is more than twice as high as typically required to effectively treat autoimmune hyperthyroidism in a patient with 20-g thyroid gland and will result in a high radiation exposure that is medically unjustified and not “as low as reasonably achievable (ALARA)” [13]. Administration of fixed activities without any adjustment for the mass to be treated and the expected kinetics is not recommended.

### Definition of Graves’ disease, toxic nodular goiter, toxic multinodular goiter

Hyperthyroidism can present as either persistent subclinical or overt thyrotoxicosis due to excessive synthesis and blood secretion of thyroid hormone(s) by either the whole thyroid gland or hyperfunctioning nodule(s) [1, 9].

Graves’ disease is autoimmune hyperthyroidism due to the presence of stimulating autoantibodies against the TSH receptor (TRAb) that promote thyroid growth and hyperfunction, frequently presenting as overt hyperthyroidism in adults living in iodine sufficient areas [1, 27, 28].

Conversely, the presence of hyperfunctioning thyroid nodule(s) (i.e., TNG or TMNG) is a frequent cause of hyperthyroidism in adults from non-iodine sufficient areas. Somatic gene mutations (i.e., TSH-R gene) have been described in TNG or TMNG, and the most common clinical presentation is subclinical hyperthyroidism [1]. Nevertheless, cardiovascular morbidity and mortality are increased in patients with TNG/TMNG despite the subclinical (or subtle) form of hyperthyroidism frequently encountered in such cases.

### Diagnostic approaches and alternative treatments

Nuclear medicine physicians are overall responsible for the delivery of RAI therapy to patients affected by hyperthyroidism and non-toxic goiter.

A detailed personal and family history and an accurate clinical examination are mandatory [1]. GD patients are frequently young or young adults with a family history or past medical history of other autoimmune diseases (e.g., rheumatoid arthritis, vitiligo, coeliac disease) [1, 29–31].

Non-autoimmune hyperthyroidism is typically diagnosed in older patients [9, 27, 32, 33]. In particular, TMNG patients frequently have a history of nontoxic goiter for many years or decades preceding hyperthyroidism onset. As a consequence of low iodine intake in regions with iodine insufficiency, chronic TSH stimulation can produce a progressive increase in size and number of nodules which may develop autonomous growth and function over time.

Accordingly, hyperthyroidism progresses gradually from subclinical to overt hyperthyroidism, frequently triggered by an iodine overload event (e.g., amiodarone treatment, iodinated contrast administration) [27, 32, 34].

Information about the current or prior use of substances able to reduce RAI uptake into the gland should be carefully obtained (Table 1]. Young patients with GD mainly exhibit symptoms of sympathetic activation (e.g., anxiety, hyperactivity, heat intolerance, and tremor), while older patients present more frequently with unexplained weight loss and cardiovascular symptoms/signs [27, 35]. Additionally, GD may be characterized by the involvement of peripheral tissues [27, 28, 34, 36, 37]. Graves’ orbitopathy (GO) is the most common and serious extra-thyroidal manifestation, affecting up to 50% of GD patients [27, 37]. GO may be present at the initial diagnosis of thyroid autoimmune disease, or may slowly develop after its onset [37].

Diagnosis of autoimmune hyperthyroidism relies on biochemical testing for anti-thyroid receptor antibody (TRAb) (99% sensitivity and specificity) which represents the diagnostic hallmark of GD. In TNG/TMNG patients, a T3-toxicosis (i.e., low/suppressed TSH with high FT3 and normal FT4) frequently represents the earliest stage of hyperthyroidism, while thyroid autoantibodies are usually absent [9, 27, 28, 32].

Thyroid ultrasonography (TUS) represents a useful tool for imaging evaluation of both GD and TNG/TMNG patients. In GD patients, TUS frequently demonstrates an enlarged gland with different ultrasonographic patterns, such as multiple hypoechoic areas, or diffuse hypoechoic gland, with or without nodules, with an important increase of vascularization on color flow Doppler, which helps distinguish between GD and destructive thyroiditis, particularly when radionuclide imaging is contraindicated, such as during pregnancy and lactation [9, 32]. In non-autoimmune patients, TUS provides information regarding nodule size, echographic features (frequently hyperfunctioning nodule is isoechoic with respect to the surrounding parenchyma), and vascular mapping of the nodule(s) on color flow Doppler evaluation.

Thyroid scintigraphy (TS) performed using Na[^99m^Tc]TcO_4_ or Na[^123^I]I is recommended for evaluation of patients with low-normal serum TSH either for identifying hyperfunctioning nodule(s) and assessing the degree of suppression of extranodular thyroid parenchyma [1, 8, 9, 27, 32, 38] or if TRAb is negative and/or GD diagnosis is unclear. Moreover, Na[^131^I]I uptake (RAIU) is required for proceeding with dosimetry-guided RAI therapy. RAIU is usually elevated in patients with GD while thyroid scintigraphy shows diffusely increased radiotracer activity throughout both thyroid lobes [9, 27, 32]. RAIU values can be either normal or high in TNG/TMNG patients, while thyroid scintigraphy shows heterogenous radiotracer activity throughout the thyroid gland [1].

Fine needle cytology (FNC) is suggested for excluding malignancy for nodules with suspicious features at TUS (e.g., hypoechoic, blurred/irregular margins, microcalcifications (< 2 mm), taller than wide) [39] and hypofunctioning (i.e., cold nodule) at thyroid scintigraphy. TUS should always include imaging evaluation of the cervical lymph nodes [40–42]*.*

The medical treatment of autoimmune and non-autoimmune hyperthyroidism with anti-thyroid drugs (ATD; i.e., thionamides, carbimazole, methimazole, and propylthiouracil) is aimed to restore euthyroidism and can be used as a first-line treatment (achieving long-term remission in approximately 30% of GD cases) or to prepare patients before RAI therapy or surgery for reducing the risk of early side effects (mainly in elderly patients or in those with cardiovascular comorbidities) [6, 9, 42].

Finally, surgery which depending on the precise condition can be either a hemithyroidectomy or total thyroidectomy could be the first choice in (i) women planning a pregnancy in < 6 months, (ii) patients with symptomatic compression or large goiters (≥ 80 g), (iii) patients with low RAIU, (iv) patients with documented or suspected thyroid malignancy (including patients with nodules cytologically indeterminate) or large (≥ 4 cm) non-functioning thyroid nodule, and (v) patients with moderate to severe active GO [9]. In GD patients with moderate to severe GO, some authors suggested performing a rhTSH-aided RAI therapy after thyroidectomy for obtaining a total thyroid ablation. This would be more effective than thyroidectomy alone for improving GO [43, 44].

### Radioiodine therapy in autoimmune and non-autoimmune hyperthyroid patients

#### Patient facility

RAI treatment delivery depends on national legislation regarding the therapeutic use of Na[^131^I]I. In many countries, RAI therapy is performed in an outpatient setting, reserving an inpatient facility for patients affected by important comorbidities and/or in whom severe complications can be expected, and/or according to Na[^131^I]I activity administrated and local legislation. Finally, an inpatient facility should be preferred in cases of bladder or bowel incontinence since it allows for safe disposal of excreta.

RAI therapy must be performed by a trained nuclear medicine physician in the nuclear medicine unit, with the availability of nursing and medical physics support (mainly if a dosimetric approach is planned).

#### Patient preparation, information, and instructions

Adequate preparation before performing RAI therapy is very important for reducing the risk of early side effects, such as exacerbation of thyrotoxicosis (i.e., transient rise in FT3 and FT4 levels) due to radiation-induced thyroiditis, occurring in about 10% of all treated patients or, more rarely, the so-called “thyroid storm” (more common in GD patients with poorly controlled hyperthyroidism) [9, 10, 45, 46]. Thus, patients should be ideally treated in a euthyroid state (overt hyperthyroidism should be avoided mainly in patients with active GO). Accordingly, ATD should be stopped 2–5 days before RAI therapy. However, longer withdrawal periods (2–8 weeks) are advised when propylthiouracil (PTU) is used due to its more pronounced radioprotective effect on thyroid tissue than methimazole’s [1, 6, 47–51]. However, high cure rates were reported after short PTU withdrawal before RAI therapy in patients treated with personalized dosimetric approaches [52]. Using a dosimetry strategy with an intended absorbed dose of 250 Gy (and achieved dose > 200 Gy) excellent ablative results for GD were obtained with only a 2-day PTU withdrawal period before RAIU testing and subsequent RAI therapy delivery [52].

Performing RAI therapy in euthyroid status may be more important in patients with overt hyperthyroidism and/or in elderly patients and/or in patients with comorbidities (e.g., cardiovascular, cerebrovascular, systemic, or pulmonary hypertension, renal failure) [9, 10, 45, 46, 53–55] for avoiding increased heart rate (due to the so-called T3-toxicosis) and possible, even if rare, supraventricular tachycardia including atrial fibrillation or flutter [9].

If possible, ATD medication should not be used in the days/weeks after RAI therapy administration since it can reduce therapy efficacy [10]. However, in elderly patients with pre-existing heart disease, even if asymptomatic, post-therapy use of ATD(s) could be considered starting 3–7 days after RAI therapy delivery [9].

Patient preparation before administration of RAI therapy includes discontinuation of iodine-containing products (e.g., toothpaste, disinfectant, hair dye) since they can reduce RAIU and, as per consequence, the success rate of RAI therapy.

RAI therapy must be postponed for at least 6 months in patients who received amiodarone treatment, and for several weeks to months for patients who received radiographic contrast agent, depending on the type of substance used, e.g., 6–8 weeks for water-soluble agents, vs. 3–6 months for lipophilic agents. In such patients, RAIU is obligatory before RAI therapy [11, 12] while an iodine urine measurement could be useful as well [6]. However, it is not available in everyday clinical practice of many nuclear medicine departments.

The ß-adrenergic blockade should be used both before and after RAI therapy in patients with severe and symptomatic hyperthyroidism, especially in elderly patients and those with cardiovascular disease, for avoiding side effects such as tachycardia or cardiac arrhythmia [6, 56, 57].

In NTG/TMNG patients, RAI therapy should be postponed if serum TSH is high-normal or elevated for avoiding the effect of RAI on normal thyroid tissue since it increases the risk of developing hypothyroidism [9, 58].

In GD patients with active and mild GO and without risk factors like high TRAb level (based on institutional assay and thresholds) and/or smoking, RAI therapy without steroid therapy should be considered as an acceptable approach mainly in patients with comorbidities such as diabetes and/or osteoporosis. Steroid therapy is recommended in such patients if risk factors are present [9]. In addition, in GD patients with active moderate to severe GO in whom RAI therapy is chosen since other treatments are not feasible, steroid treatment is recommended for minimizing the risk of GO worsening, mainly if concomitant risk factors (e.g., smoking) are present [1, 9, 59]. In such patients, intravenous administration of steroids is recommended using a starting dose of 0.5 g once weekly for 6 weeks, followed by 0.25 g once weekly for 6 weeks [58, 59].

For patients referred for RAI therapy who are at high risk of development/worsening of GD orbitopathy, prophylactic steroids oral administration is recommended using a daily dose of 0.2 mg prednisone (prednisolone)/kg body weight for 6 weeks after RAI therapy [59] or alternatively 0.3–0.5 mg/kg/bodyweight as an initial dose, subsequently gradually tapered and discontinued [58].

Steroid therapy was debated for a long time [60] but is not recommended for patients with present but inactive GO, and non-smoking GD patients without apparent GO by current EUGOGO recommendations [58]. Steroid therapy should not be started before RAI therapy being able to reduce RAIU and consequently the efficacy of the treatment [61]. Accordingly, steroid therapy should be started 1–3 days after RAI therapy and continued for several weeks [1, 4–6, 9].

Lithium therapy is not frequently used in hyperthyroid patients. However, being able to lengthen ^131^I half-life in the gland (without reducing iodine uptake), it may be used in GD patients with low (< 20%) RAIU starting immediately after RAI therapy and continued until 7 days post-therapy. Presently, its clinical significance on RAI therapy effectiveness is not completely clear even if some literature data showed that adjuvant short-term (7 days) use of lithium (900 mg/day) increases RAI therapy efficacy without an increase in side effects (e.g., orbitopathy worsening in GD patients) [62, 63]*.* Since side effects are currently reported in about 10% of patients, the use of lithium should be evaluated case by case and considered only if RAI therapy is not feasible without it, and alternative treatment options are not available or not feasible [6].

In general, no special diet is strictly required before RAIU testing and subsequent RAI therapy administration. However, it seems sensible to avoid iodinated salt and reduce iodine-rich food (e.g., seaweeds, fish, milk, and eggs), respectively, for at least 7 days before RAIU [1]. In this light, an iodine urine measurement may be useful in selected cases (i.e., patients whose diet is mainly based on seaweed or is rich in supplements containing iodide) [9].

Pregnancy is an absolute contraindication for RAI treatment. In all fertile females, it can be excluded by obtaining hCG serum testing within 72 h before RAI administration. If there is a suspicion of early pregnancy (not detectable by beta-HCG) according to anamnestic data, RAI therapy should be postponed. Finally, pregnancy must be avoided for almost 4 months after RAI therapy [64]. Breastfeeding must have been discontinued for at least 6 weeks before RAI therapy administration for allowing time for mammary tissue involution with the goal of avoiding high radioiodine uptake and, consequently inadvertent radiation to hypertrophied mammary gland tissue [6, 9, 11, 65].

The nuclear medicine physician must provide verbal and written information to patients prior to therapy administration, addressing the following:

(i) Aim and strategy of RAI therapy;
(ii) Final functional outcome including the risk of persistent/recurrent disease with possible subsequent re-treatment;
(iii) Medical therapy to use before and/or after RAI therapy;
(iv) Possible early and/or late side effects and their possible treatments;
(v) Radiation precaution instructions according to national laws
(vi) Clinical management, laboratory tests, and imaging studies during follow-up.

The patient’s written informed consent must be obtained before RAI therapy administration.

#### Procedure and Na[^131^I]I activity

Na[^131^I]I is preferentially administered orally as a capsule since it allows an easier and safer treatment than using the liquid form or intravenous administration, thus reducing the risk of contamination. Intravenous administration can be used in patients with parenteral nutrition, vomiting, or other causes of the inability to process iodine through the gastrointestinal tract. The liquid form can be used in patients with severe swallowing difficulties; on the other hand, oral administration is not feasible in some countries due to legislative compliance, and therefore, intravenous administration may be required even for patients with swallowing difficulties. In addition, liquid Na[^131^I]I is generally supplied for intravenous injection, and therefore, administering liquid orally would be classed as an unlicensed way of administering Na[^131^I]I (off-label) with different absorbed doses to the mouth. If oral administration is chosen, as happen in most cases, patients have to be fasting overnight before RAI therapy and 1–2 h after treatment. Following RAI therapy administration, patients should be encouraged to drink a sufficient quantity of fluid for 24–48 h for reducing the radiation absorbed dose to the bladder (critical organ) and minimize body radiation exposure by enhancing urinary RAI excretion.

The administered Na[^131^I]I therapeutic activity depends both on the aim (e.g., ablative vs. functional therapy in GD patients) and the strategy (fixed-activity method vs. dosimetric approach) of RAI treatment.

Accordingly, the patients’ restrictions regarding work and with respect to family members and the general population will be different. In general, patients have to be advised to increase the distance and reduce the contact time when interacting with others (mainly children, pregnant, and breastfeeding women) for reducing radiation exposure that in Europe should not exceed 1 mSv cumulative dose per year [6, 13, 64, 66–68].

## RAI therapy for patients with autoimmune hyperthyroidism (Graves’ disease)

### Introduction

Graves’ disease (GD) is the most common cause of persistent hyperthyroidism in adults living in iodine-sufficient regions and is more prevalent among smokers [1]. GD patients mostly suffer from overt hyperthyroidism.

### Definition and epidemiology

GD has an autoimmune etiology due to the loss of immune tolerance to thyroid self-antigens with consequent production of organ-specific auto-antibodies targeted against the TSH receptor (TSH-R) expressed on thyrocyte membrane, the so-called TRAb [27, 28].

GD is the most common form of hyperthyroidism in iodine-sufficient areas accounting for about 80% of all cases. Autoimmune hyperthyroidism has a peak incidence between 30 and 60 years of age, with a higher prevalence in women (1:5–7) [27, 28, 33, 34]. The annual incidence of GD is estimated at 20 − 50 cases per 100,000 individuals [27, 28].

### Indication and contraindications (absolute and relative)

- RAI therapy is considered the first line treatment for GD patients when:
  (i) There are contraindications to the use of an anti-thyroid drug (ATD);
  (ii) Increased surgical risk is documented (e.g., heart failure, laryngeal nerve palsy, pulmonary hypertension)
  (iii) Recurrent hyperthyroidism occurs after ATD withdrawal or sub-optimal thyroid surgery;
  (iv) Patients are elderly with comorbidities;
  (v) Patients do not have access to a high volume thyroid surgeon (especially in pediatric patients);
  (vi) Thyroid remnant tissue remained and malignant GO occurred/persisted after surgery [6, 9, 69].
- RAI therapy may be considered a first line treatment for GD patients when:
  (i) They are > 10 years, regardless of gender, having small to medium goiter and inactive GO;
  (ii) They are > 10 years, regardless of gender, having small to medium goiter and low to mild active GO; Conversely, RAI therapy is contraindicated for the following GD patients:
    1. Absolute contraindications:
      1. Pregnant or breastfeeding patients
      2. Patients with the (multi)-nodular variant of GD with suspected or confirmed thyroid cancer
      3. patients ≤ 5 years old
    2. Relative contraindications:
      1. Patients with very large goiters (≥ 80 g)
      2. Patients with active moderate-severe GO and high TRAb levels (mainly if smokers) [9, 69–75].

In such cases, a careful consideration of pros and cons of RAI therapy, surgery, and long-term ATD treatment is advised, taking into account local resources and patients’ preferences, in order to customize patients’ management.

RAI therapy should be delayed as much as possible in children between 5 and 10 years of age [1, 9].

### Concepts for RAI therapy delivery in Graves’ disease: functional vs. ablative

There are 2 concepts for RAI therapy delivery in GD patients:

- The functional dose concept in which the aim of RAI therapy is to correct subclinical or overt hyperthyroidism with the goal of reaching euthyroidism as soon as possible.
- The ablative dose concept in which the aim of RAI therapy is to achieve hypothyroidism as soon as possible, based on evidence that the definitive success rate in controlling hyperthyroidism is much higher than that obtained with the functional dose concept (> 90% vs. < 70%, respectively) [ 6, 9, 52, 76, 77]. In addition, ablative RAI therapy reduces the volume of the gland correcting both mechanical issues (e.g., dyspnea, dysphagia) and aesthetic features of the neck [ 78].

RAI therapy success rate depends on both radioiodine-administered activity (and its kinetics, i.e., uptake and effective half-life in the gland) and thyroid volume [6, 9, 49, 50, 79, 80]. A recent meta-analysis showed that the delivered radiation absorbed dose to the thyroid is directly related to outcome [7].

A thyroid radiation-absorbed dose of 150 Gy is necessary to obtain euthyroidism according to the functional dose concept [6, 14, 75], while it is necessary to deliver a radiation-absorbed dose to the thyroid ranging between 200 and 300 Gy according to the ablative dose concept [6, 14, 76, 81] (Table 2]. The frequency of persistent hyperthyroidism is very low (8%) when a thyroidal radiation-absorbed dose of approximately 300 Gy is delivered [1, 76].

**Table 2:** A summary of the intended absorbed dose with respect to thyroid disorders and purpose of RAI therapy

Disease	Absorbed dose	Aim
GD	100–150 Gy	Normalize thyroid function (functional dose concept)
GD	200–300 Gy	Hypothyroidism and replacement (ablative dose concept)
TNG	300–400 Gy	Normalize thyroid function (functional dose concept)
TMNG	150–300 Gy	Normalize thyroid function (functional dose concept)

To date, the unsatisfactory long-term outcome of GD patients treated according to the functional dose concept resulting in a higher incidence of recurrent hyperthyroidism and the risk of possible GO worsening is the main reason for the currently preferred ablative dose concept [6, 77]. Accordingly, our panel members favor the implementation of the ablative dose concept for all GD patients.

In the recently published European Thyroid Association guidelines on the management of GD in pediatric patients [82], the ablative dose concept was also advised as the strategy of choice in pediatric patients since it reduces the risks of both persistent/recurrent hyperthyroidism and delayed radiogenic-induced thyroid neoplasms within residual non-ablated thyroid tissue [6, 83–87].

#### Fixed-activity method

The administration of fixed RAI activity is the easiest and most commonly used method for performing RAI therapy in GD patients for the definitive treatment of hyperthyroidism.

The fixed-activity method was originally based on the administration of a fixed activity to all patients. Moreover it may be refined by estimating the thyroid gland size by palpation, morphological, or functional imaging (i.e., TUS or thyroid scintigraphy, respectively) (i.e., fixed administered activity per mass unit) [1, 6]. In addition, Na[^131^I]I thyroid gland maximum uptake and turnover should be empirically taken into account when therapeutic RAI activity is planned [88].

Most often, fixed activities between 370 and 555 MBq are employed to treat GD patients [1, 9, 11, 75]. However, higher activities may be required when the thyroid volume is higher than 40 ml. In this setting, a dosimetric approach should be considered [89].

In daily clinical routine, the main advantage of the fixed-activity method is the simplicity of pre-treatment logistics. However, a potential disadvantage of the fixed-activity method is the potential risk of over-treatment, associated with unnecessarily high radiation exposure to the patient, or under-treatment with a higher cumulative incidence of persistent/recurrent hyperthyroidism [1, 78, 90, 91]. Notably, however, neither randomized clinical trials nor retrospective comparison studies demonstrated the inferiority of the fixed-activity approach compared to dosimetric approach.

#### Dosimetric approach

Results of studies reporting the relationship between the thyroid radiation absorbed dose and treatment outcome are conflicting [92]. However, a multicentric prospective randomized trial [93] and a systematic review and meta-analysis including the most relevant retrospective studies [7] on RAI therapy of Graves’ disease with complete dosimetry showed that there is a significant dependence of treatment success rates on the actual thyroid radiation absorbed dose. According to the combined analysis [7], the delivery of 150 Gy, 200 Gy, and 300 Gy to the thyroid gland is expected to eliminate hyperthyroidism in 74%, 81%, and 88% of cases, and restore euthyroidism in 38%, 35%, and 29% of cases, respectively. Besides the thyroidal radiation absorbed dose, a larger size of the thyroid gland (often associated with fast iodine kinetics) appears to be an independent risk factor for residual disease [76, 93, 94].

A target dose of at least 200 Gy is recommended for the definitive treatment of patients with refractory Graves’ disease. Up to 300 Gy is appropriate especially for large glands and very pronounced hyperthyroidism if a more reliable therapeutic effect is desired in the short term. If restoration of a euthyroid state is reasonably possible in milder disease with a low risk of recurrence and is desired by the patient, approximately 150 Gy target dose should be aimed for. The success of the therapy is not guaranteed due to uncertainties in determining the therapeutic RAI activity and individual variables that have not yet been sufficiently investigated. Patients should be informed that treatments with lower target radiation absorbed dose are more likely to require retreatment and that the likelihood of hypothyroidism requiring lifelong thyroid hormone replacement increases with an escalation of the target radiation absorbed dose. Even if euthyroidism is successfully restored after treatment, regular long-term monitoring of hormonal status remains necessary because the rate of hypothyroidism increases continuously over years following RAI therapy.

If the therapeutic RAI activity is estimated from an early uptake measurement according to Eq. (3], an estimate of the effective half-life of *T*_est_ = 5.5 days suggests that approximately 6, 8, and 12 MBq multiplied by the mass in gram and divided by the 24-h Na[^131^I]I uptake (RAIU) are to be administered for target doses of 150, 200, and 300 Gy, respectively.

Example: A patient with 35-g thyroid mass and 65% 24-h Na[^131^I]I uptake, RAIU(t = 24 h) = 0.65, who is to be treated with a thyroid absorbed dose of 200 Gy, should be given Aa = 8·35/0.65 MBq = 430 MBq. In the case of fast iodine kinetics, recognizable by an early and high iodine retention and high Technetium Thyroid Uptake (TcTU) in the Na[^99m^Tc]TcO_4_ scintigram, a shorter half-life in the tissue and therefore underestimation of the absorbed dose is to be expected. Especially with large thyroid glands, which might be less sensitive to radiation, this can lead to high failure rates [94] justifying to aim for absorbed doses higher than 200 Gy.

If a pre-therapeutic assessment of the kinetics is omitted, activity dosage should be linear to the mass M assuming that about half of the administered activity decays in the gland, corresponding, e.g., to iodine kinetics with 79%, 67%, or 57% 24-h Na[^131^I]I uptake and 4.5, 5.5, or 6.5 days, respectively, the effective half-life of the activity in the gland. The therapeutic activity is calculated as 9, 12, and 18 MBq multiplied by the mass in grams for target doses of about 150, 200, and 300 Gy, respectively. Example: A thyroid gland with 20-g mass is to be treated with Aa = 9·20 MBq = 180 MBq for an absorbed dose of 150 Gy.

## RAI therapy for patients affected by non-autoimmune toxic nodular goiter/toxic multinodular goiter

### Introduction

Hyperfunctioning thyroid nodules — either isolated (TNG) or in the context of a multinodular goiter (TMNG) — represent approximately 5–10% of all thyroid nodules, and they are the most common cause of hyperthyroidism in elderly patients who come from iodine-deficient areas [38, 95–97]. The degree of hyperfunction is variable in such nodules. Accordingly, they can produce either sub-clinical or overt hyperthyroidism [98–102].

### Definition and epidemiology

A somatic activating mutation affecting the thyrotropin receptor (TSHR) gene and the gene encoding for the stimulatory GPT-binding protein (Gsα) have been reported as the main cause of TNG and have also been described in many TMNG patients [1, 103–105].

In iodine deficiency areas, TNG and TMNG represent the main cause of hyperthyroidism accounting for approximately 60% of all cases while their prevalence significantly decreases in the iodine-sufficient areas (e.g., USA) being 15–30% of hyperthyroidism cases [106].

### Indications and contraindications (absolute and relative)

RAI therapy is a safe and effective therapeutic option for the definitive treatment of hyperthyroidism in patients affected by TNG or TMNG [1, 107].

RAI therapy is considered the first-line treatment option for patients affected by subclinical or overt hyperthyroidism in the following circumstances: (i) a solitary hyperfunctioning nodule (i.e., Plummer disease); (ii) one or multiple hyperfunctioning nodules in multinodular goiter without suspected or confirmed thyroid cancer; (iii) advanced age; (iv) comorbidities (e.g., cardiovascular, cerebrovascular, systemic, or pulmonary hypertension) resulting in higher surgical risks; (v) previous history of neck surgery and/or irradiation; (vi) limited or no access to a high volume thyroid surgeon [1, 9].

Conversely, RAI therapy is contraindicated for the following TNG or TMNG patients:

I. Absolute contraindications:
  1. Pregnancy or breastfeeding
  2. Suspected or confirmed thyroid cancer
II. Relative contraindications:
  1. Large TMNG
  2. Compressive neck symptoms and/or signs [1, 9].

### RAI therapy for non-autoimmune toxic nodular goiter /toxic multinodular goiter

In TNG or TMNG patients, the first goal of RAI therapy is to correct subclinical or overt hyperthyroidism, preferably restoring euthyroidism. The success of RAI therapy is mainly related to radioiodine-administered activity and its kinetics in TNG or TMNG and nodule(s) volume [1, 9, 108].

In this light, even if some authors suggested lower absorbed doses in the past [109] an absorbed dose to the target(s) ranging from 150 to 300 Gy is currently recommended to correct hyperthyroidism [6, 110–113], while higher absorbed doses (i.e., up to 400 Gy) were reported to slightly improve RAI therapy success rate [113] (Table 2].

Overall, the success rate is very high ranging from 81 to 94% of TMNG and TNG patients, respectively [1, 6, 9, 78, 110–113]. The risk of persistent or recurrent hyperthyroidism is higher in TMNG than in TNG patients, reaching up to 20% of all treated patients [1, 9, 78, 114–116]. Conversely, the risk of developing hypothyroidism is higher for patients > 45 years old and/or treated with higher radioiodine activities and/or with higher RAIU and/or partial suppression of extra toxic nodule(s) parenchyma and/or pre-treated with ATD [1, 9, 117, 118]. The incidence of hypothyroidism after RAI therapy ranges between 20 and 60% depending on disease (TNG or TMNG), degree of extra-nodular suppression, and length of follow-up [1, 90, 92, 112, 117–121].

The second aim of RAI therapy in such patients, mainly in those with larger goiters, is to reduce the volume of hyperfunctioning nodule(s) and/or goiter (if extra-nodular thyroid parenchyma is not suppressed), thus alleviating mechanical complaints (e.g., dysphagia, dyspnea) that affect a significant number of such patients. Toxic thyroid nodule(s) volume reduction is 35% within 3 months and up to 45% after 24 months following RAI therapy administration [1, 78, 114, 122].

#### Fixed-activity method

As for GD patients, the administration of predefined fixed RAI therapeutic activities is the easiest and most commonly used method for the delivery of RAI therapy in TNG and TMNG patients.

According to this method, the estimation of hyperfunctioning nodule(s) volume is obtained by palpation or preferably by TUS or TS [1, 6] and ^131^I nodule(s) maximum uptake and turnover (evaluated by RAIU trend) should be considered for RAI therapy planning.

However, the fixed-activity method has limited accuracy [1, 91], and consequently, the cumulative incidence of persistent/recurrent hyperthyroidism (mainly in TMNG patients) or, conversely, of hypothyroidism (mainly in TNG patients whose extranodular parenchyma is not suppressed) is not negligible [1, 78, 90, 118].

Most often, fixed activities ranging from 370 to 740 MBq are used to treat TNG or TMNG patients [1, 9, 11].

RAI therapy efficacy is limited when hyperfunctioning nodule(s) volume is > 26 ml. In such patients, a dosimetric approach is considered more useful for obtaining an effective treatment [89].

The fixed-dose method offers the same advantages and limitations already discussed for RAI therapy in GD patients.

#### Dosimetric approach

In non-autoimmune toxic goiter, dosimetry should ideally only take into account the kinetics in the autonomously functioning tissue, whereby not all, but only part of the thyroid gland parenchyma is affected, typically in the form of one or more nodules. With uptake probes usually used to measure activity kinetics [14], dosimetry is reliably possible if the mass of the autonomic tissue can be measured and the healthy thyroid tissue is completely suppressed. Underdosing and the consequent risk of persistent or recurrent hyperthyroidism occurs when a non-negligible fraction of the measured activity is located in unsuppressed healthy tissue or autonomously functioning tissue outside of the nodules taken into account in the mass determination. The main risks for developing hypofunction are incomplete suppression of the remainder of the gland and a high targeted absorbed dose in a large target volume. In toxic goiter with a single or only a few reliably measurable nodules, RAI treatment based on complete dosimetry of the autonomously functioning volume eliminates hyperthyroidism in 85–100% of individuals treated with 300–400 Gy target dose with rates of hypothyroidism typically between 10 and 20% [112, 113].

When calculating the target dose for the treatment of toxic nodule, the measured iodine uptake must be corrected by the nodule/whole thyroid uptake ratio obtained from the scintigram. The target dose for the nodule is 300–400 Gy.

When the estimation of a target mass is not possible, absorbed doses of 100–150 Gy to the mass of the entire gland should be applied to calculate the therapeutic RAI activity [92, 110, 112].

If complete dosimetry is omitted, activity dosing should be based on an early uptake measurement, as indicated by a meta-analysis [123]. Equation (3] suggests administering approximately 4, 6, 8, 12, or 16 MBq multiplied by the mass in gram and divided by the 24-h Na[^131^I]I uptake for target doses of 100, 150, 200, 300, or 400 Gy, respectively. The suggested value of 5.5 days for *T*_est_ is about 10% less than the mean effective half-lives typically observed in most toxic nodular goiters [110, 113] but limits the potential error to ± 25% for the vast majority of individuals [124]. Examples: A patient with a 10-g nodule with 30% 24-h Na[^131^I]I uptake should be administered Aa = 12·10/0.3 MBq = 400 MBq for a nodule absorbed dose of 300 Gy. Another patient with multinodular goiter in a gland with 30-g total mass and 40% 24-h Na[^131^I]I uptake needs Aa = 6·30/0.4 MBq = 450 MBq for a mean thyroid absorbed dose of 150 Gy.

The dosimetric approach following the European standard operational procedure provides the most accurate results regarding achieved and intended target doses. The Marinelli formula seems to enable minimal deviations of achieved doses from target doses in TMNG, although other individualized strategies taking into account at least the thyroid volume also result in low deviations of presumed achieved dose from the intended dose [14, 125].

## RAI therapy for patients with non-toxic goiter

### Introduction

RAI therapy has been used for the volumetric reduction of goiter and/or thyroid nodule(s) in patients with benign non-toxic nodular goiter (NTG) for the past 30 years [92, 126, 127]. The current guidelines support the use of RAI therapy as an alternative to thyroid surgery, particularly for patients considered at high surgical risk due to significant comorbidities, or in patients who wish to avoid surgery [3, 126, 128, 129].

### Definition and epidemiology

NTG is defined as thyroid gland enlargement, with or without intrathyroidal nodules, associated with TSH, FT3, and FT4 values within the reference range.

It is one of the most prevalent thyroid disorders, particularly in iodine-insufficient regions, with a worldwide population health impact.

Thyroid nodules represent a very common clinical presentation, being diagnosed by clinical evaluation and/or high-resolution TUS in up to 60–70% of the overall population, with increased prevalence in female patients and the elderly [9, 102, 130–132].

The incidence of NTG in iodine-sufficient areas is about 0.1/350.000 new diagnoses per year. However, the incidence is higher (2/350.000 per year) in the population that underwent radiation exposure of the neck [133].

### Indications and contraindications for RAI therapy in NTG

RAI therapy is indicated for NTG patients with compressive symptoms and a total thyroid volume < 100 ml, particularly if elderly and/or with comorbidities (e.g., cardiovascular) and/or with a history of previous neck surgery [126]. In addition, RAI therapy is indicated for the treatment of large NTG with intrathoracic extension when surgery cannot be performed or is refused by the patient [126].

Of course, the choice to treat such patients using RAI depends on the local expertise, radiation regulations, and patient’s preference [126].

Absolute contraindications to RAI therapy include the following: (i) pregnancy or lactation (however such patients are generally elderly); (ii) cases with suspected or confirmed thyroid cancer, and (iii) patients in whom a fast resolution of tracheal compression is required. Relative contraindications to RAI therapy include (i) patients with 24 h RAIU value < 15% (however, rhTSH may be selectively used in such cases), (ii) thyroid volume > 100 ml; (iii) when the nodule responsible for symptoms (e.g., dyspnea and/or dysphagia) is scintigraphically hypofunctional/cold [126].

### RAI therapy approach for NTG patients

Since clinical, diagnostic, and therapeutic management of NTG patients is controversial, nuclear physicians must perform an accurate patient selection for RAI therapy taking into consideration, the risk of treatment failure, goiter recurrence/persistence, and patient’s preferences. Accordingly, a detailed personal and familiar history, clinical examination, laboratory tests, and imaging evaluation are needed.

Thyroid ultrasonography is used to obtain information regarding nodule(s) size and topography within the thyroid gland, echographic features, and vascular mapping on color flow Doppler evaluation. In addition, TUS-guided FNC is a fundamental tool for ruling out malignancy [9].

Second-level morphological imaging (i.e., CT without contrast or MRI) should be considered in NTG patients with (very)-large goiter and/or with intrathoracic extension for whom high-resolution three-dimensional visualization of the thyroid is required to calculate gland volume and to evaluate its relationship with the surrounding anatomical structures [134]. CT or MR-based estimations of the smallest cross-sectional tracheal area permit comparison between baseline and post-therapeutic measurements to assess RAI therapy results [134–138]. However, the agreement between CT and MR in evaluating both thyroid gland volume and the smallest cross-sectional tracheal area is not known. As per consequence, the same imaging modality should be used before and after RAI therapy [134].

Thyroid scintigraphy performed using Na[^99m^Tc]TcO_4_ or Na[^123^I]I is indicated for patients with low-normal serum TSH (especially in iodine deficiency areas) both for excluding hyperfunctioning nodule(s) and for identifying cold nodule(s) and their topography within the gland [102], and for evaluating radiotracer distribution heterogeneity throughout the thyroid gland, which may decrease RAI therapy efficacy [122, 134, 139]. RAIU is a fundamental diagnostic tool for the evaluation of NTG patients since the prescribed therapeutic RAI activity should be adjusted according to RAIU values [74, 102, 122].

Currently, no worldwide consensus exists regarding the management of NTG patients recognizing that no ideal treatment is available. As per consequence, more than 30% of clinicians prefer an observational approach avoiding any type of treatment, especially for patients with mild or moderate compressive symptoms in whom malignancy has been ruled out [2–4, 102, 134].

Iodine supplementation has been suggested as an adequate and feasible approach for the prevention of NTG since goiter development is strongly associated with iodine deficiency, even if mild [134, 140]. However, there is limited information regarding its efficacy in patients with already established goiter, while its use has been associated with an increased incidence of Hashimoto’s thyroiditis and papillary thyroid carcinoma [134, 141]. However, these possible effects are overlooked regarding the routine use of iodine supplementation in a large part of Europe and in North America [3, 4, 134].

Levothyroxine therapy is generally the preferred choice of many members of both the European Thyroid Association and the American Thyroid Association (ATA) [3, 4]. However, there is no agreement regarding its efficacy, and several authors have demonstrated that a sufficient goiter reduction can be observed in only a limited number of patients [134, 142–144]. In particular, goiter reduction observed in patients on levothyroxine therapy is lower than that observed in patients who underwent RAI therapy [140]. Moreover, the gland volume tends to return to baseline values when levothyroxine therapy is discontinued [142].

Finally, for obtaining goiter reduction, levothyroxine therapy aimed at suppressing the serum TSH level is often prescribed resulting in sub-clinical or overt hyperthyroidism, which should be avoided, especially in elderly (> 60 years) patients due to untoward side effects on the cardiovascular and skeleton systems [134, 145, 146].

Based on the above considerations, levothyroxine therapy should be suggested only in young NTG patients with modestly increased thyroid volume [134].

Surgical removal of the thyroid gland [especially (near)-total-thyroidectomy] should be the preferred therapy in selected NTG patients (see the specific paragraph). The main advantage of surgery is related to the immediate and significant goiter reduction with prompt relief of compressive symptoms and histopathological analysis of the thyroid tissue [134].

However, some potential, permanent or temporary, side effects must be taken into account when the surgical approach is planned [134, 143, 147]. In particular, recurrent laryngeal nerve palsy, hypoparathyroidism, respiratory complications, and tracheomalacia can occur in a not negligible number of patients, mainly in those with larger NTG with substernal extension [134, 148–152].

In addition, goiter recurrence can be observed in NTG patients who underwent less than subtotal thyroidectomy [134, 153–156]. In such patients, RAI therapy represents a favorable alternative to reoperation [134, 153–156] for avoiding the risk of permanent vocal cord paralysis and/or hypoparathyroidism that is 3-to-tenfold higher than at initial operation [134, 148, 149, 157].

For reducing the risk of surgical complications, thyroid surgery should be always performed by expert surgeons in high-volume centers [102].

### RAI therapy delivery for treatment of non-toxic goiter

The main goal of RAI therapy in NTG is to reduce the volume of the thyroid gland [3, 92, 126, 128, 129]. Following RAI therapy administration goiter size volumetric reduction of approximately 40–50% after 1 year, and up to 60% after 2–5 years has been reported [92, 122, 126, 138, 144, 158–165]. The response to RAI therapy is individual and may be different among patients but symptoms most often improve and patient satisfaction is high [126, 138, 160, 165, 166].

No consensus exists regarding the prescribed therapeutic RAI activity for the treatment of NTG [92, 126, 167]. Based on published clinical experiences, a therapeutic RAI activity ranging from 3.7 to 14 MBq/g thyroid tissue corrected for RAIU has been recommended, to impart 100–175 Gy radiation absorbed dose to the thyroid tissue [122, 126, 138, 158, 159, 161–165]. Certainly, the most pronounced thyroid volume reduction rate was observed when the highest thyroid radiation absorbed dose (i.e., 175 Gy) was used [126, 168], but this approach increases the whole-body radiation exposure [126, 169].

However, RAI therapy efficacy is inversely correlated to initial NTG volume. As per consequence, in patients with very large NTG (i.e., > 100 ml) in whom RAI therapy is chosen as the preferred treatment, the volume reduction rate is around 35% after 1 year [92, 122, 126]. Likewise, efficacy is reduced in NTG patients with low RAIU values [92, 126].

Repeated courses of RAI therapy have been reported to result in significantly larger rates of goiter reduction in patients with very large goiters, and this option should be considered especially for patients unable or unwilling to undergo reductive thyroid surgery. Furthermore, while resolving cosmetic concerns may take a more extensive volumetric reduction, quite often the reduction in volume seen after a single RAI therapy in very large goiters is still sufficient to alleviate compressive complaints to such an extent that a second RAI therapy is obviated.

#### Patient facility

The patient facility can be different according to national legislation on the therapeutic use of ^131^I. Please refer to the paragraph on RAI therapy for hyperthyroidism.

#### Patient preparation, information, and instruction

As per hyperthyroid patients, iodine-containing products such as administration of amiodarone or radiographic contrast agents should be discontinued well in advance of therapy, or better avoided altogether since they reduce RAIU and, consequently, RAI therapy efficacy can be decreased [1, 6, 9–12]. Patients with (very)-large NTNG (≥ 100 ml) and severe trachea narrowing (especially if < 1 cm) should always be treated under steroid coverage (e.g., prednisolone, 25 mg daily for 14 days) [6, 126]. Moreover, consideration for inpatient RAI therapy, mainly for elderly patients with comorbidities (e.g., heart failure), will ensure a hospital setting for addressing possible post-treatment complications [6, 144, 158, 170].

Patient preparation, logistical information, and instructions for the delivery of RAI therapy for NTG patients are similar to hyperthyroid patients regarding:

(i) The advice to avoid/reduce the intake of iodized salt, seaweed, iodine-containing nutritional supplements, and iodine-rich foods such as sea fish, milk, and eggs for at least 7 days before RAIU and subsequent RAI therapy [1]. Urinary iodine measurement should be performed before RAIU/RAI therapy in patients whose diet is mainly based on seaweed or is rich in iodide-containing nutritional supplements [9];
(ii) Pregnancy is an absolute contraindication for RAI treatment. In all fertile females, it can be excluded by obtaining hCG serum testing within 72 h before RAI administration. If there is a suspicion of early pregnancy (not detectable by beta-HCG) according to anamnestic data, RAI therapy should be postponed. In addition, pregnancy must be avoided for 4–6 months after RAI therapy, while breastfeeding must be discontinued for at least 6 weeks before RAI therapy administration for avoiding high radioiodine uptake and, consequently, radiation absorbed dose to the breasts [6, 11].
(iii) The instructions that nuclear medicine physicians must provide to patients before RAI therapy.
(iv) The requirement for obtaining written informed consent from patients.

#### Procedure and Na[^131^I]I activity

As for hyperthyroid patients, Na[^131^I]I is preferentially administered orally in the form of a capsule but liquid form or intravenous administration can be used in patients with severe swallowing difficulties or patients with parental nutrition, respectively.

For rapid 131-I absorption, it is recommended that oral RAI therapy is administered on an empty stomach and this condition must be maintained in the following 1–2 h.

To reduce the radiation absorbed dose to critical organs and diminish radiation exposure to the entire body, patients should be encouraged to drink a large quantity of fluid for at least 24–48 h following RAI therapy.

The prescribed Na[^131^I]I therapeutic activity largely depends on the target thyroid volume, the RAIU values, and radioiodine distribution in the gland.

Accordingly, the instructions regarding patients’ restrictions on work, with family members, and with respect to the general public will vary depending on the amount of prescribed radioactivity.

Prior to RAI therapy administration all patients must receive detailed information for reducing both radiation exposure and contamination risk to others, particularly to children, pregnant, and breastfeeding women [6, 13, 64, 66, 67,68].

#### Fixed-activity method

The easiest and most common method used for performing RAI therapy in NTNG patients is the administration of fixed RAI activities.

According to this method, the estimation of the target treatment volume is obtained by palpation and/or TUS and/or CT or MR (in cases with sub-sternal extension), functional mapping of the goiter is obtained by TS, and RAIU testing should be performed for RAI therapy planning [1, 6, 136, 160, 171–178].

There is no consensus regarding the fixed RAI activity prescribed for NTG treatment and a wide range of RAI activities have been reported [92, 126, 136, 160, 171–179]. Most often, fixed activities ranging from 600 to 1110 MBq are used to treat NTNG patients [126, 136, 159, 160, 171–177].

#### Dosimetric approach

Although the volumes of euthyroid goiters often are substantially increased, the biokinetics usually corresponds approximately to those of healthy thyroids with normal or only moderately increased radioiodine uptake and a long half-life within the gland [168]. After pre-therapeutic dosimetry, treatment should target a mean thyroid absorbed dose of about 100 Gy [134], in selected cases up to 150 Gy. An often-used activity of 3.7 MBq multiplied by the mass in gram and divided by the 24-h ^131^I uptake administers a mean thyroid absorbed dose of 120 Gy to a large goiter treated for size reduction if the effective half-life of the activity in the gland is 7 days.

The dosimetric approach following the European standard operational procedure provides the most accurate results regarding achieved and intended target doses [14, 125].

#### rhTSH-aided RAI therapy for NTG

Although rhTSH use was initially restricted to thyroid cancer patients, it has been used since the early 2000s to increase thyroidal RAIU in patients with NTG [173, 180, 181]. Administration of rhTSH is associated with goiter reduction enhancement by 35–55% as compared with Na[^131^I]I therapy without rhTSH stimulation. The adjuvant role of rhTSH in RAI therapy for NTG has been studied mainly for patients with very large goiters and in those with a low baseline RAIU [179]. However, rhTSH is not yet approved for this indication, and administration in a patient with NTG represents an off-label use. Therefore, the current guidelines cannot endorse the general use of rhTSH for the treatment of NTG, unless rhTSH-aided RAI therapy is regulated according to local rules (e.g., by authorization of the local Ethical Committee) and cautiously chosen individually for each patient in view of the risks, benefits, experience of the treating physician, and patient’s personal preference.

Declining efficacy with increasing goiter size and low thyroidal RAIU are the major challenges in conventional RAI therapy for NTG, and these factors represent the main indications for rhTSH-stimulated RAI therapy [182]. The potential benefits of rhTSH-aided RAI therapy are based on the marked increase in thyroidal RAIU, resulting in increased radiation absorbed dose to the thyroid without increasing the administered RAI activity (superiority strategy) or reduction of the administered RAI activity while maintaining the same thyroidal radiation absorbed dose (dose-reduction or equality strategy) [183]. According to published literature, rhTSH should be administrated 24 h before RAI therapy (optimal time interval) rather than 2 h, 48 h, or 72 h for maximizing the thyroidal RAIU increase [92, 126, 168, 184]; however, no consensus exists about the dose of rhTSH for aiding RAI therapy. Although in most studies a dose of 0.1–0.3 mg has been given, a wide range of doses (i.e., 0.005–0.9 mg) have been used [92, 126, 174, 185]. However, the choice of rhTSH dose to use for aiding RAI therapy is strongly related to the purpose of the treatment. Indeed, a dose of 0.1 mg is recommended in the equality strategy, whereas a higher rhTSH dose is employed (i.e., 0.3 mg) coupled with higher prescribed therapeutic RAI activity in the superiority strategy [92], resulting in a higher goiter reduction as compared to the equality approach [92]. All in all, based on the present knowledge the optimal rhTSH dose for aiding RAI therapy is most likely in the range of 0.03–0.1 mg. Indeed, in this dose range, a significant improvement of the thyroidal RAIU is obtained while minimizing the risk of side effects [126].

The rationale for using rhTSH-aided RAI therapy in NTG is to obtain an enhanced goiter volume reduction (GVR) which is maintained for several years after therapy with the goal of reducing the need for additional therapy, such as a second RAI treatment or thyroid surgery. However, little is known about the long-term risk of goiter recurrence after RAI therapy, whether with or without rhTSH stimulation [186]. Reported adverse events include administered RAI activity-dependent increased risk of thyroid hyperfunction and acute thyroid swelling when RAI therapy is preceded by rhTSH stimulation. While other serious acute side effects seem of little concern when using low rhTSH doses, the more pronounced goiter reduction, seen with rhTSH-stimulated RAI therapy, is unfortunately achieved at the expense of an up to fivefold increase in the rate of permanent hypothyroidism [175, 182].

Pretreatment with a single, low rhTSH dose allows a reduction of the prescribed RAI activity in patients with nodular goiter and significantly enhances goiter volume reduction in some studies. Notably, however, available evidences do not support its routine clinical use, and the rhTSH is not EMA-approved for this indication. Its out-of-label use requires strict compliance with local laws and recommendations under the responsibility of nuclear medicine physicians.

## RAI therapy in pediatric patients

### Special considerations for RAI therapy in children

There are special considerations regarding RAI therapy in pediatric patients, especially in pre-pubertal children who are much more susceptible to the harmful effects of ionizing radiation. Furthermore, the developing body of pediatric patients requires consideration of body size before adapting RAI therapy concepts that were designed for fully-grown adults. The special considerations applicable to pediatric RAI therapy are discussed below:

#### Disease spectrum

Autonomous nodules or obstructive goiter are very rare in children; nearly all patients referred for RAI therapy have been diagnosed with GD refractory to medical therapy with ATD. Therefore, unless explicitly stated otherwise, the recommendations and considerations in this section will pertain to GD.

#### Age

The reluctance for employing RAI therapy in pediatric GD increases with decreasing patient’s age. According to the recently published European Thyroid Association Guidelines [82] on the treatment of Graves’ disease in children and adolescents, Na[^131^I]I can theoretically be used in any patient with GD. However, a very young age (< 5 years; because of a greater long-term theoretical risk of malignancy) is seen as a near-absolute contraindication. Age 5–10 years is regarded as a relative contraindication. Interestingly, an update in this guideline is a recommendation that ATD should be maintained for multiple years in pediatric GD patients so that rather than being referred for surgery, children with a near-absolute or relative age-based contraindication for RAI therapy patients can often be kept on ATD until they reach an age adequate for undergoing RAI therapy.

#### Patient preparation

Other than the standard preparations as described for adults (e.g., stopping ATD), no special medical preparations are necessary for pediatric patients. Of course, depending on the age of the patient and the possibilities allowed by local radiation protection regulations, it may be possible or even necessary to allow a parent to be co-hospitalized with the patient after appropriate preparation of the parent/caregiver.

### Therapeutic concepts

For pediatric GD patients, the aim of RAI therapy is (functional) thyroid ablation in order to minimize the risk of GD recurrence and possible future malignant transformation of persistent, viable cells with radiogenic-induced genetic damage. To achieve this aim, a number of concepts have been described in pediatric populations, including a fixed activity approach with activities ranging from 200 to 800 MBq [6], a weight-based approach of 15 MBq Na[^131^I]I per gram thyroid tissue (as estimated by ultrasound) and a dosimetric concept aiming at a delivered dose to the thyroid of at least 300 Gy [6, 12].

There is no definitive literature evidence demonstrating the superiority of one of these concepts in a pediatric population, although a recent systematic review in adults was able to clearly demonstrate that higher delivered radiation absorbed doses are associated with higher rates of successful functional thyroid ablation [7]. Although fixed-activity concepts have been demonstrated to be successful, they do carry a risk of over-treatment: the activity given might be higher than necessary for achieving an ablative dose to the thyroid, exposing non-target tissue to unwarranted high radiation doses, which is particularly unjustifiable in pediatric patients. Conversely, there is also a risk of undertreatment, thus exposing non-target tissues to radiation whilst not achieving the therapeutic goal. Therefore, for optimizing the chance of success, while at the same time minimizing excessive radiation exposure to non-target tissues, we recommend performing thyroid dosimetry if available. This recommendation also applies to the treatment of autonomous nodules in children.

If hyperthyroidism persists after the first RAI therapy course, repeat RAI treatment after a sufficiently long interval (i.e., 3–6 months) is possible, similar to adult patients.

### Side effects in pediatric patients

Side effects following RAI treatment in pediatric patient population are very rare and include occasional mild neck tenderness over the thyroid area in the first-week post-RAI therapy administration. Notwithstanding comparatively high administered RAI activities, for RAI therapy aimed at functional thyroid ablation (i.e., hypothyroidism) in pediatric patients, observational studies found no evidence of increased risk of malignancy or fertility problems after almost 40 years of clinical follow-up [86, 187–189].

### Alternative treatment

For pediatric patients, the selection of RAI therapy versus total thyroidectomy is often a contentious choice. Local opinion and expertise will also play a role. Of course, each pediatric case necessitates interdisciplinary consultation including a pediatric endocrinologist, thyroid surgeon, and nuclear medicine physician specialized in thyroid disease. The definitive treatment choice involves shared decision-making with the patient and the parents/legal guardians. An absolute or relative contraindication for surgery or RAI therapy such as very young age, pregnancy, or a markedly elevated risk of perioperative morbidity may direct the choice of treatment modality. Surgery remains the preferred definitive treatment option for GD patients < 10 years of age, patients with other (relative) contraindications for RAI therapy, those with a large or nodular goiter, and those with active Graves’ ophthalmopathy necessitating quick complete removal of thyroid tissue.

## Adverse effects of RAI therapy

Although RAI therapy is safe and usually well-tolerated, either acute or late side effects may occur, mainly in patients with uncontrolled severe hyperthyroidism and/or active Graves’ orbitopathy [1, 92]. However, patients with overt and uncontrolled hyperthyroidism should not be treated, while those with severe active thyroid orbitopathy should be firstly considered for surgery treatment [1, 190].

The main side effects are summarized in Table 3 and briefly reported below.

**Table 3:** Summary of early and late side effects

Side effect	Onset	Pathophysiology	Symptoms
Thyroid swelling	Early	Inflammatory reaction to irradiation	Thyroid pain Sensation of thyroid growth Dyspnea in patients with a large goiter
Radiation thyroiditis and post-therapy thyrotoxicosis	Early	Transient rise in fT3 and fT4 levels	Exacerbation of hyperthyroidism symptoms Thyroid storm (rare): -high fever -central nervous system manifestations, gastrointestinal and hepatic manifestations -heart failure
Radioiodine-Induced sialadenitis	Early/late	Concentration in salivary of iodide due to the sodium iodine symporter expression	Swelling, Periductal pressure Duct constriction Pain Xerostomia Taste dysfunctions
Immunogenic effects	Early	Release of thyroid antigens from destroyed follicular cells with increase of TRAb,	RAI therapy causes the transient increase of TRAb
Hypothyroidism: transient or persistent	Late	Transient hypothyroidism: unclear cause Persistent hypothyroidism: Thyroid irradiation	Transient hypothyroidism: no symptoms or signs, only biochemical Persistent hypothyroidism: Typical sign of hypothyroidism
Graves’ orbitopathy	Late	B-cells and macrophages activation with cytokines secretion	Worsening or appearance of orbitopathy

### Early side effects

Early side effects can occur either promptly or during the first weeks after RAI therapy. In general, they are especially correlated to initial thyroid volume and hyperthyroid status before RAI administration.

#### Thyroid swelling

Thyroid swelling is an early side effect that can be observed in patients with large toxic or, more frequently, non-toxic goiter. Patients could manifest thyroid pain and sensation of anterior neck discomfort due to the inflammation process caused by irradiation of thyroid tissue (i.e., radiation-induced thyroiditis). There are rare reported cases of elderly patients with very-large nodular goiters experiencing rapid thyroid enlargement due to edema which may cause tracheal compression and dyspnea. This possible side effect should always be considered, particularly when rhTSH-aided RAI therapy is planned in patients with large NTG or patients with known tracheomalacia [1, 160].

The aforementioned symptoms are generally mild or moderate. As per consequence, medical therapy is not usually required or may be considered to alleviate local pain symptoms (e.g., paracetamol). However, in patients with higher risk factors (e.g., larger goiter size), they can be prevented/treated using a non-steroidal anti-inflammatory agent for 24–48 h after RAI administration while corticosteroids should only be used in selected patients since it can reduce RAI therapy efficacy [1, 61].

#### Radiation thyroiditis and post-therapy thyrotoxicosis

The prevalence of radiation thyroiditis is very low with about 1% of all treated patients. It may occur during the first weeks after RAI therapy and can produce a transient rise of serum thyroid hormone levels due to follicular cell damage.

As per consequence, a mild to a severe worsening of thyrotoxicosis may occur in about 10% of patients, mainly among those with poorly controlled hyperthyroidism before RAI therapy [54]. Conversely, the so-called thyroid storm is a rare but life-threatening condition that requires immediate diagnosis, comprehensive, and advanced medical treatment using anti-thyroid drugs (ATDs), inorganic iodide, beta-adrenergic receptor antagonists, corticosteroids, and antipyretic drugs [191] and, if all else fails, emergency thyroidectomy.

A careful selection of the optimal time point for performing RAI therapy and correct patients’ premedication are mandatory for avoiding/reducing the risk of thyrotoxicosis worsening and, especially, thyroid storm condition [1].

#### Radioiodine-induced sialadenitis

Although it is a more frequent complication in case of RAI therapy for differentiated thyroid cancer, radioiodine-induced sialadenitis can also occur in hyperthyroid patients, especially if treated with a high RAI activity (e.g., ≥ 1000 MBq), both as an acute and a late side effect [1]. Iodine is concentrated in the salivary gland by the NIS and then secreted into saliva. During this process, salivary glands are exposed to dose-dependent radiation damage [192]. Clinical symptoms include salivary gland swelling (frequently transient), local pain, xerostomia, or dysgeusia (taste dysfunction), which may occur in as many as 20–30% of patients [192, 193]. The use of lemon juice and/or salivation-inducing snacks (like lemon/orange candy), starting 24 h following RAI therapy is a valid radioprotective procedure to diminish RAI damage to salivary parenchyma [1, 194–196].

#### Immunogenic effects

The so-called Marine-Lenhart syndrome [197] is a rare side effect of RAI treatment observed in TNG patients during the first days after RAI therapy, with a reported prevalence ranging from 2.7 to 4.1% [198, 199]. However, its incidence is significantly higher in patients with elevated serum levels of thyroid peroxidase antibody (TPOAb) before RAI therapy, as well as in patients who became TPOAb positive after RAI therapy [198, 200–203]. These observations support the hypothesis that immunogenic hyperthyroidism in such patients represents an exacerbation of a pre-existing and occult immunogenic thyroid disorder [1].

RAI administration, as well as surgery, could produce a transient increase of TRAb due to the release of thyroid antigens from damaged follicular cells. As per consequence, autoimmune hyperthyroidism can be observed in such patients, but no correlation has been found between serum TRAb rise and RAI activity administered [1, 204].

The most serious consequence of immunogenic side effects is the activation or new onset of Graves’ orbitopathy (GO) [92].

### Late side effects

#### Hypothyroidism: transient or persistent

Persistent hypothyroidism can be counted among side effects of RAI therapy for TNG or NTNG patients [1, 92]. However, in these patients, thyroid surgery will produce hypothyroidism too [92]. Conversely, hypothyroidism represents the optimal result (i.e., main goal) when RAI therapy is performed in GD patients according to the newest ablative dose concept [1, 6, 9, 52, 76, 77]. However, some patients experience transient hypothyroidism that may manifest 2–5 months after RAI therapy and spontaneously recover within a few months without the development of symptoms or signs of hypothyroidism [1, 205]. Differentiating between transient and permanent hypothyroidism in the early months following RAI therapy remains a clinical conundrum that requires adequate clinical and laboratory follow-up to establish the optimal treatment plan, especially for GD patients with orbitopathy [1, 206, 207].

#### Graves’ orbitopathy

Several studies demonstrated a significant association between RAI therapy and the development or worsening of GO [1, 208]. In a systematic review that included nine studies comprising a total of 1773 patients, Li et al. reported that RAI therapy is a significant risk for new-onset or worsened orbitopathy as compared to ATD treatment (*p* < 0.00001), while prophylactic administration of steroids has been demonstrated to be an effective method for preventing this event (*p* = 0.002), especially in patients with moderate orbitopathy [208]. High TRAb levels before RAI administration, smoking, and uncorrected post-treatment hypothyroidism are associated with a more aggressive orbitopathy and a worse prognosis [70]. For this latter reason, thyroid function should be always evaluated in the first weeks after RAI therapy to avoid the risk of development or worsening of GO due to uncorrected hypothyroidism. However, early (i.e., after 2 weeks following RAI therapy) levothyroxine substitutive treatment has been already proposed for preventing hypothyroidism [207]. In GD patients at high risk for the development/worsening of orbitopathy following RAI therapy, the administration of prophylactic oral steroids is recommended using a daily dose of 0.2 mg prednisone/kg body weight for 6 weeks after RAI therapy [59]. Conversely, intravenous administration of steroids is recommended in GD patients with moderate to severe active GO using a starting dose of 0.5 g once weekly for 6 weeks, followed by 0.25 g once weekly for 6 weeks [59].

#### Cancer incidence

There is no convincing evidence for an increased risk of secondary cancers and/or increased mortality in patients treated by RAI therapy [209–211].

## Pregnancy following RAI treatment

Women should be recommended to avoid pregnancy 4–6 months after radioiodine treatment for benign thyroid disease, mainly to ensure to avoid pregnancy in uncontrolled hypothyroidism while radioprotection concerns are limited in such cases.

Specific counseling about pregnancy and fatherhood should be provided to patients planning a pregnancy after RAI therapy. Strict communication between the patients, the referring physician, and the nuclear medicine physician is pivotal to provide the best service.

## Data Availability

The data that support the present guideline are available on request from the first author (acampenni@unime.it).
